# ADE-CycleGAN: A Detail Enhanced Image Dehazing CycleGAN Network

**DOI:** 10.3390/s23063294

**Published:** 2023-03-21

**Authors:** Bingnan Yan, Zhaozhao Yang, Huizhu Sun, Conghui Wang

**Affiliations:** School of Electronic Engineering, Xi’an Shiyou University, Xi’an 710065, China

**Keywords:** image defogging, CycleGAN, Dep residual blocks, multi-head attention

## Abstract

The preservation of image details in the defogging process is still one key challenge in the field of deep learning. The network uses the generation of confrontation loss and cyclic consistency loss to ensure that the generated defog image is similar to the original image, but it cannot retain the details of the image. To this end, we propose a detail enhanced image CycleGAN to retain the detail information during the process of defogging. Firstly, the algorithm uses the CycleGAN network as the basic framework and combines the U-Net network’s idea with this framework to extract visual information features in different spaces of the image in multiple parallel branches, and it introduces Dep residual blocks to learn deeper feature information. Secondly, a multi-head attention mechanism is introduced in the generator to strengthen the expressive ability of features and balance the deviation produced by the same attention mechanism. Finally, experiments are carried out on the public data set D-Hazy. Compared with the CycleGAN network, the network structure of this paper improves the SSIM and PSNR of the image dehazing effect by 12.2% and 8.1% compared with the network and can retain image dehazing details.

## 1. Introduction

Deep learning is widely used in outdoor scenarios such as automatic driving, personnel detection and industrial data detection. At present, the mainstream deep learning system is based on the recognition of clear pictures. However, in hazy weather, the pictures collected are blurred, which reduces the recognition and detection accuracy of deep learning algorithms. Therefore, dehazing research plays an important role in improving the clarity of the picture, and is also one of the hotspots of many scholars’ research.

The current mainstream single image dehazing method is divided into two categories: the restoration method based on the atmospheric light model and the dehazing method based on deep learning. The dark channel prior method of He [1] and the color attenuation method of Zhu [2] are representative image defogging methods which are based on physical models and image information. Although these methods have good defogging performance, the dark channel prior theory cannot achieve the expected defogging effect when the brightness scene is similar to the current illumination brightness, and Zhu’s color attenuation method can remove most of the fog, but there still exists fog in part of the enhanced image. In the research of deep learning dehazing, Cai [3] first proposed the DehazeNet network and used the convolutional neural network instead of the artificial prior method to realize the end-to-end mapping relationship between the foggy image and the transmission image constructed from a single image, but this method affects the dehazing effect of the network to some extent by estimating the intensity of the transmission map and atmospheric light. Ren [4] proposed a multi-scale MSCNN network, which predicts the refractive index of the overall image and refines the local area by using two different scales of the CNN network to learn the transmission of the image features, but the resulting image has some problems in blurred edge details. Li [5] proposed the AOD Net network structure, through combining the transmission map and atmospheric light refractive index as a parameter for training. However, after dehazing, the image is dark and distorted.

Image defogging algorithms have always been a research difficulty in the field of object detection image preprocessing, for which researchers have carried out a lot of research. Zhang [6] put forward the end-to-end dense connected pyramid defogging network. Based on the atmospheric scattering model, the defogging image is achieved by using two networks to estimate the transmittance and atmospheric light, respectively, and according to the proposed joint discriminator based on the generation countermeasure network framework, judge whether the defogging image is effective. Dong [7] used the back projection technology to design the dense feature fusion module and proposed a multi-scale enhanced defogging network to solve the problem of spatial information. Mehta [8] proposed a generation countermeasure network for hyperspectral guided image defogging and built the network structure of HideGAN by designing an enhanced version of CycleGAN named R2HCycle and an enhanced version of GAN named H2RGAN; Mehta used HideGAN to combine the hyperspectral image with the cycle consistency and skeleton loss and analyze the relevant information carried by the entire spectrum to defog the degraded image. Qin [9] combined the channel attention mechanism with the pixel attention mechanism and proposed an end-to-end feature fusion attention network of a feature attention (FA) module, basic block structure and attention based feature fusion (FFA) structure of different levels to realize image defogging. Although the image after defogging is excellent in texture details and color fidelity, there is still some haze left.

Jaisurya [10] proposed an enhanced CycleGAN architecture for unpaired single-image dehazing with an attention-based transformer architecture embedded in the generator. He [11] proposed ACC-GAN to maximize the mutual information between the hazy and haze-free domains. In the latent representation space, a contrastive constraint is introduced to ensure that the restored image is closer to the clear image and farther away from the blurred image, thereby indirectly regulating the unsupervised dehazing process. Ma [12] proposes an attention-based non-paired image learning method for single image dehazing. This method leverages CycleGAN’s constrained transfer learning capabilities and recurrent structure to perform unsupervised image dehazing tasks on unpaired data.

CycleGAN (Cycle-Consistent Generative Adversarial Networks) can complete training without pairing data set information [13,14,15]. Moreover, CycleGAN proposes an end-to-end dehazing model, which can learn the mapping relationship between foggy images and fog-free clear images, but it cannot guarantee that the foggy images are mapped to the desired high-quality clear images, and there is image blur after dehazing and information loss issues. 

In response to the above problems, we use CycleGAN as the basic model framework and propose a detail enhanced image dehazing CycleGAN network (ADE-CycleGAN). By combining the idea of the U-Net [16] network, we added the depth feature extraction and fusion module to improve the ability to extract features and added a multi-head attention mechanism to make up for the limitations of the single attention mechanism in the network, thereby effectively improving the dehazing effect of the network.

The main contributions of this paper include three aspects:

(1) Dep residual blocks were constructed in multiple parallel branches of the generator to extract the depth feature information of the image, and the branches were fused with the supersampled feature information to increase the details of the reconstructed image and avoid the distortion and blur of the restored image.
(2) We added a multi-head attention mechanism, which could extract feature information from multiple dimensions and balance the deviation generated by the same attention mechanism in different directions, thereby effectively improving the overall defogging effect of the network.
(3) To solve the problem of image detail loss and color change, we added circular perception loss and color adjustment loss [17] on the basis of the loss function, retained the details of the original image and improved the effect of the generator to generate the dehazing images.

## 2. Related Work

### 2.1. Attention Mechanism

In foggy weather, it is difficult for conventional networks to capture interested targets. Therefore, scholars propose attention mechanisms [18] to strengthen the network model to increase the degree of attention to target areas and increase the overall network feature extraction ability. The attention mechanism is essentially a resource allocation scheme, which increases the degree of attention to the target area by allocating different weight coefficients.

The attention mechanism is mainly divided into the channel attention mechanism and the spatial attention mechanism. Jie [19] proposed a variant of the channel attention mechanism: SE attention mechanism. This method gives different weights to different positions of the image through the angle of the channel domain, obtains more important feature information, strengthens the features of each channel and improves the feature expression effect of the network. Woo [20] proposed a new attention mechanism called CBAM (convolutional block attention module), which integrates the channel attention mechanism with spatial attention mechanism. This method connects the channel attention mechanism with the spatial attention mechanism to obtain more comprehensive and reliable attention information and allocates the attention of the two dimensions, which improves the connection of each feature in the channel and space, and it can also extract the effective characteristics of the target. 

In recent years, the attention mechanism [21,22,23,24,25] has the advantage of focusing on the areas. Moreover, the attention mechanism has become the theoretical basis of deep learning fields such as image recognition and image segmentation. However, a single attention mechanism can only establish a dependency relationship between the query and key (i.e., give a query, calculate the correlation between the query and key, and then find the most appropriate value according to the correlation) and cannot establish a dependency relationship between itself and the whole. At the same time, the same attention mechanism will also produce partial and overall deviation, which has certain limitations.

### 2.2. Depth Separable Convolution

The depth separable convolution [26] has lighter parameters than the convolution. The idea of deeply separable convolution is to decompose the original standard convolution operation into multiple channels by channel convolutions and a 1 × 1 point-by-point convolution operation, and the original standard convolution layer is decomposed into two different convolution layers, in which one convolution core of the channel-by-channel convolution layer is responsible for one channel, and one channel is only convolved by one convolution core. The point-by-point convolution layer is responsible for linear combinations of different channel feature maps outputs from the previous layer (the number of channels of the feature map generated in the process is exactly the same as the number of channels input), which can effectively use the feature information of different channel feature maps in the same spatial location. This decomposition can greatly reduce the amount of calculation and the number of model parameters.

## 3. Materials and Methods

Because of its convenient deployment, CycleGAN has been widely used in many industrial and intelligent transportation scenes. In comparison to GAN, there is no need for a paired data set to train and there is a good defogging effect. CycleGAN has not made too much improvement on image detail defogging, leading to the poor performance of image defogging. Therefore, it is necessary to improve the CycleGAN network to adapt to the detail defogging of the image. Considering the requirements of real-time detection, CycleGAN is selected as the follow-up improvement of this paper. The improved network structure in this paper is similar to the CycleGAN network. The ADE-CycleGAN flowchart is as follows.

### 3.1. ADE-CycleGAN

**Figure 1:**
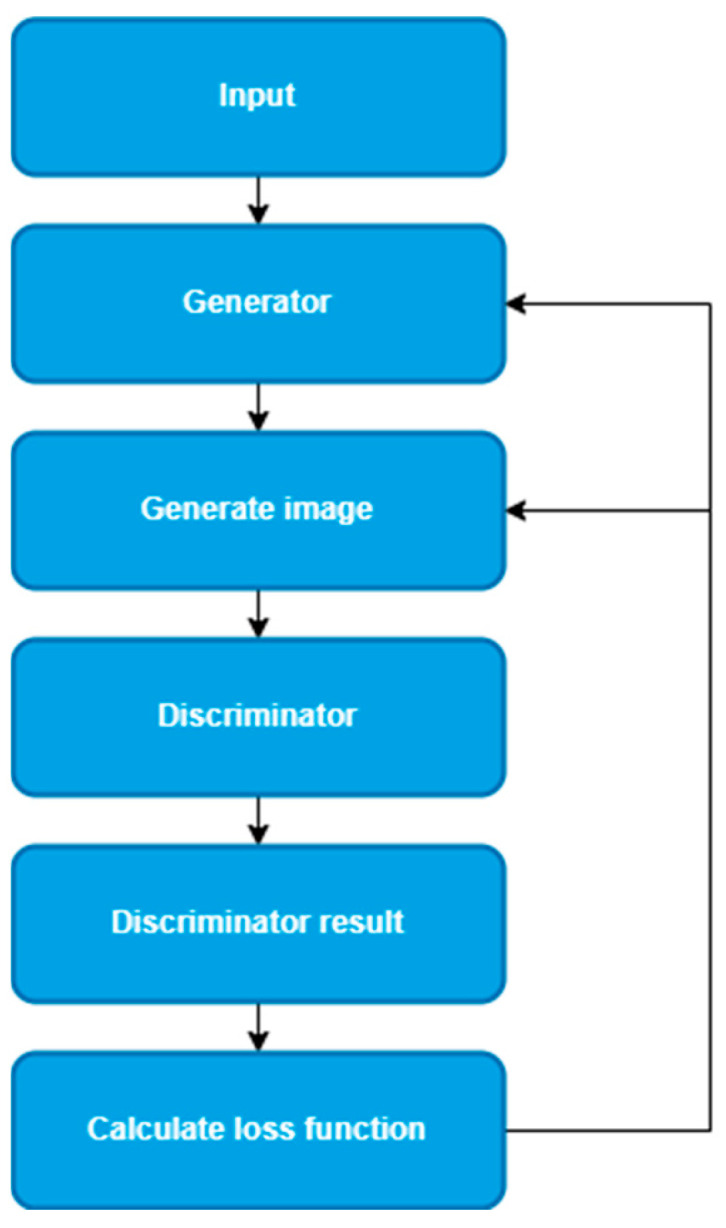
Network’s brief flowchart.

The ADE-CycleGAN demisting network architecture is shown in Figure 1, and each branch includes a discriminator and shares two generators. Firstly, input the fog image x into the generator $G_{A}$ of CycleGAN to obtain the generated image $Y^{\prime }$. Then, input the generated image $Y^{\prime }$ into the generator $G_{B}$ to obtain the generated image $x^{\prime }$. Next, input the generated image and the real clear image together into the discriminator to judge the foggy image with the discriminator and output the judgment result. Finally, according to the generated image and the discrimination results, calculate the loss function and update the parameters of the generator and discriminator to obtain the optimal defogging model.

The network architecture extracts texture data from the input images and outputs unique defrosting images. The concepts of cyclic perception loss and color adjustment loss are introduced in the loss function. By combining the two concepts, it can enhance the similarity between real images and fog-free images and improve the robustness of the network.

### 3.2. Generator Structure

In the process of image defogging, the CycleGAN network will have the problems of color change and detail loss of the resulting image [27,28,29]. In order to solve the above problems, we proposed ADE-CycleGAN (a detail enhanced image dehazing CycleGAN network). By combining the feature fusion idea with the depth Dep residual block, the generator network can better capture the detailed information in the image background, which is conducive to improving the defogging effect of the generated network, and the introduction of the multi-head attention mechanism can also improve the receptive field of the network, strengthen the expression ability of features and balance the deviation generated by the same attention mechanism.

**Figure 2:**
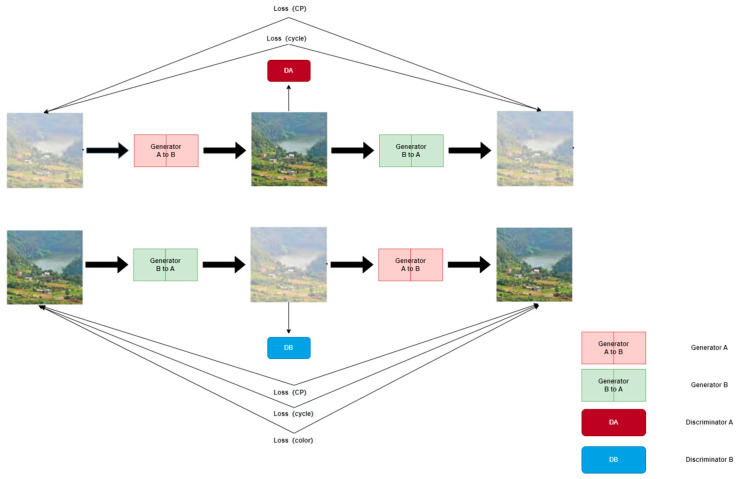
ADE-CycleGAN fog removal network structure.

The generator can preserve the contents of the background information and restore the details during the fog removal process, and its network structure is shown in Figure 2. The network structure includes a feature extraction structure, cascade residual network block, Dep deep depth block, feature fusion reconstruction structure and multi-head attention mechanism block. The convolution cores of the three convolution layers in the feature extraction structure are 7 × 7, 3 × 3 and 3 × 3. The convolution core of 7 × 7 enlarges the field of image perception in the network, and the network can better capture the details of the image. After that, two convolution cores of 3 × 3 size can effectively reduce the size of the extracted feature map, which is conducive to network training. Cascaded residual network blocks can be used to increase the depth of the generator network and thereby improve feature extraction capabilities. The Dep depth uses convolution blocks extracted from different feature diagrams and fuses them with the recovery feature diagrams sampled above as the next input; the network can effectively improve the extraction of overall and local detail information, and it can solve the problem of detail loss after image convolution and pooling reconstruction. The multi-head attention mechanism is used to determine the focus of the feature map in the model. It processes different features in different orders, providing additional flexibility in dealing with different content.

#### 3.2.1. Dep Residual Block Structure

**Figure 3:**
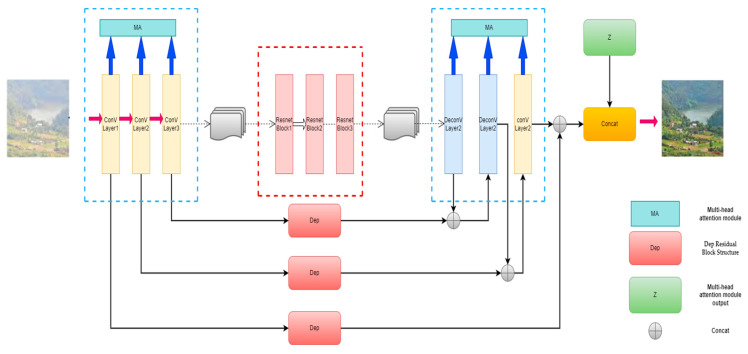
Improved generator structure.

In the process of generating images, the CycleGAN network structure can change objects and backgrounds at the same time. Taking the classic method by Zhu [28] as an example, CycleGAN changed the appearance of horses from a horse to a zebra, but it also changed the bright background to a dark background, and caused texture loss and image distortion in image detail construction. Therefore, in order to solve the problem that the detailed features disappear due to background changes during defogging and image reconstruction, we designed a Dep residual block structure in the network, which improves the network’s ability to extract feature information through a deep detachable convolution network, and increases the depth of the network to obtain a larger field of perception, so as to improve the network’s sensitivity and adaptability to similar features of different pixels. The problem of the disappearance of deep network gradients can also be solved effectively without increasing the computational complexity. The structure of the Dep residual block is shown in Figure 3.

**Figure 4:**
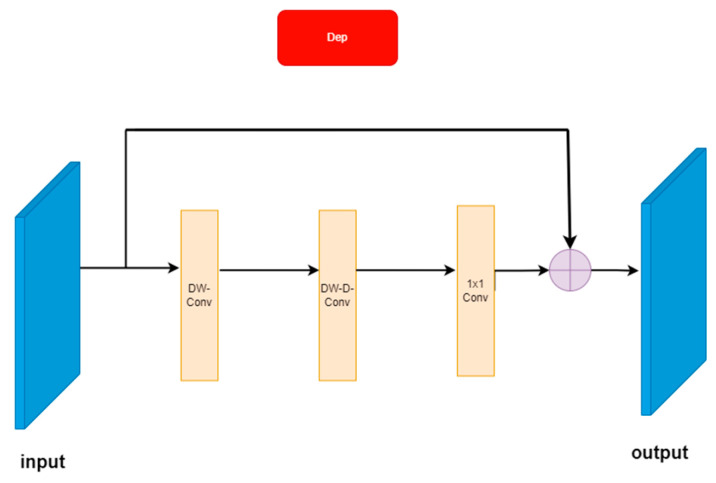
Dep residual block structure.

Using residual blocks can increase network depth; meanwhile, it also significantly increases the amount of calculation of network parameters. Thus, we introduced deeply separable convolution. It can be decomposed into two channel-by-channel convolutions and one point-by-point convolution, and it can greatly reduce the parameters of network operations while ensuring sufficient information about the characteristics. The idea is from MobileNet [30] and the feature information is extracted by 5 × 5 channel-by-channel convolution (DW-Conv). Then, the target’s receptive field is expanded by 7 × 7 channel-by-channel void convolution (DW-D-Conv) to capture multi-scale context information at the same time. Finally, the output feature map is obtained by 1 × 1 point-by-point convolution. The Dep residual blocks in Figure 4 avoided losing image feature information and transfer information to the deeper layers of the network by skipping links, enhancing the feature response of the background and image details.

(1) $$T=f_{conv}^{1\times 1}(f_{DW-D-conv}^{7\times 7}(f_{DW-conv}^{5\times 5}(X)))⊗X$$

Formula (1): $f_{conv}^{1\times 1}$ means the convolution kernel size is 1 × 1 for conventional convolution, $f_{DW-conv}^{5\times 5}$ for deep channel-by-channel convolution with a convolution core size of 5 × 5, $f_{DW-D-conv}^{7\times 7}$ for deep channel-by-channel void convolution with a convolution core size of 7 × 7 and ⊗ means the element-wise product; X represents the input feature map and T represents the new feature map after Dep feature enhancement.

#### 3.2.2. Multi-Head Attention Mechanism Fusion Structure

Multi-head attention mechanism [31] maps the query, key and value which are generated by the self-attention mechanism to multiple attention subspaces for attention calculation. It can learn the independent related information from the different representation subspaces. Combining information in each subspace can enrich the foggy pictures’ feature information. Multiple self-attention computations in a subspace yielded a number of attention matrix outputs of $Head_{n}$ and n headers, which allowed $Head_{n}$ to be stitched into the feature matrix to yield $MutilHead_{n}$.

(2) $$A_{attention}(Q,K,V)=\mathrm{softmax}(\frac{QK^{T}}{d_{k}^{1/2}})V\;head_{i}=A_{attention}(QW_{i}^{Q},KW_{i}^{K},VW_{i}^{V});i=1,2,\ldots ,h\;MutilHead_{n}=Concat(head_{1},head_{2},....,head_{n})$$

*Q*, *K* and *V* are queries, keys and values for calculating attention, $d_{k}$ represents the dimension of the key and h is the number of heads. $W_{i}^{Q}$, $W_{i}^{K}$ and $W_{i}^{V}$ is a projection matrix, which is used to project *Q*, *K* and *V* onto the i dimension.

In order to capture preferably the key information of the image, unlike the multi-head attention mechanism, which produces multiple sets of *Q*, *K* and *V* in the same feature map, this paper’s multi-head attention mechanism only produces *Q*, *K* and *V* vectors through different convolution layers. By combining *Q*, *K* and *V*, we can calculate the new multi-head attention mechanism.

**Figure 5:**
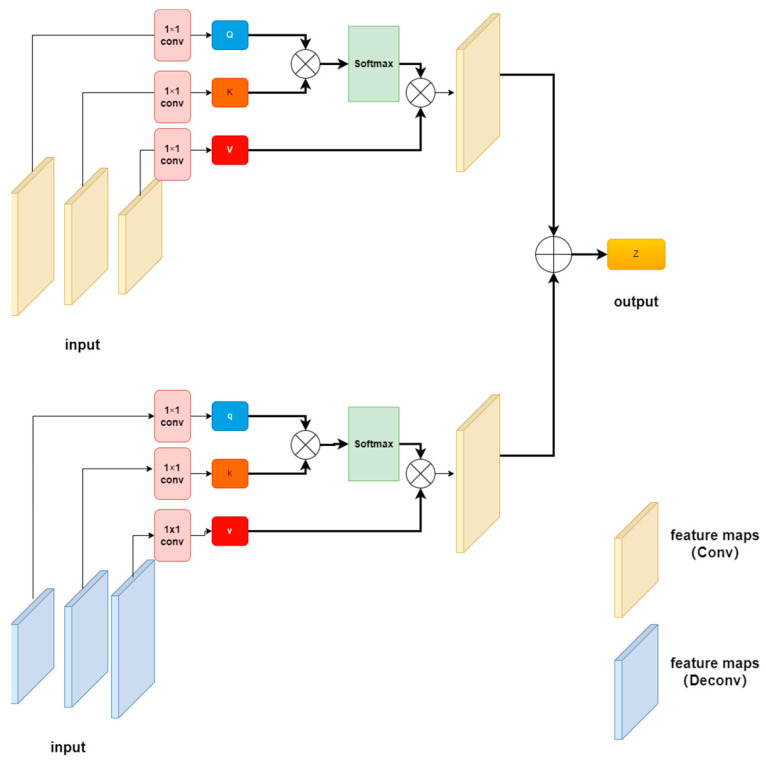
Multi-head attention mechanism module.

As shown in Figure 5, the whole module is divided into two parts: one part can calculate the attention mechanism of the feature map, while the other part calculates the same attention mechanism of the sample results, and finally splits and classifies the two. Firstly, the *Q* vector is obtained from the Convlayer1 convolution, the *K* vector is obtained from the Convlayer2 convolution and the *V* vector is obtained from the Convlayer3 convolution. Similarly, the feature map generated by DeConvlayer1 is sampled to obtain the *Q* vector through a convolution of 1 × 1, the feature map generated by DeConvlayer2 is convoluted by 1 × 1 to obtain the *K* vector and the feature map generated by DeConvlayer3 is convoluted by 1 × 1 to obtain the *V* vector for the multi-head attention weight calculation. Finally, the feature maps obtained from the two attention mechanisms are added bitwise and stitched together to form a multi-head attention model with a head number of 4.

### 3.3. Design of Loss Function

Due to the foggy image exhibiting information loss, it is difficult to recover all the texture information from the cyclic consistency loss in CycleGAN. By introducing the concept of color loss, the image to be generated and the target image can be semantically more similar. This idea is from Zisserman [28] and Wang [32]. In this paper, we added cyclic perception loss and color adjustment loss on the basis of the existing loss function. The improved loss function is as follows:

(3) $$L=Loss_{GAN}+Loss_{cycle}+Loss_{CP}+\mu Loss_{color}$$

In Formula (9), $Loss_{CP}$ is the cycle perception loss, $Loss_{color}$ is the color adjustment loss and μ is the weight factor. Cyclic perception loss can improve the semantic similarity between the generated image and the real image, while color adjustment loss can avoid serious differences between the generated image color and the original image color, and it can guide the generator to generate more realistic images.

#### 3.3.1. Cyclic Perception Loss

Due to the adversarial loss and cyclic consistency loss in the network structure, the detailed texture information of the image cannot be completely recovered. Therefore, in order to improve the quality of information recovery, the concept of circular sensing loss is introduced. Firstly, we used the second and fifth pooling layers of the pretrained VGG16 [26] network model to extract features, and used the L2 norm to calculate the features of the fuzzy image and the reconstructed fuzzy image, as well as the differences between the features of the fog-free image and the reconstructed fog-free image. The loss function is shown below. The goal of the loss function is to maintain the image structure and content characteristics during the process of deblurring and generate a more realistic image. The loss function is shown below:

(4) $$Loss_{CP}={\left\|Vgg(G_{B}(G_{A}(x)))-Vgg(x)\right\|}_{2}+{\left\|Vgg(G_{A}(G_{B}(y)))-Vgg(y)\right\|}_{2}$$

In Formula (7), $G_{A}$ and $G_{B}$ are two different generators, x is the input foggy image and y is the clear image. $G_{B}(G_{A}(x))$ and $G_{A}(G_{B}(y))$ are reconstructed graphs and *Vgg* is the feature extractor.

#### 3.3.2. Loss of Color Adjustment

The loss functions of CycleGAN often lead to changes in the brightness and contrast of the generated defog images, and even artifacts. In order to improve this reality, inspired by Wang [30], the concept of color adjustment is introduced into the overall loss function to measure the color difference between the fog-free image and the reconstructed image. Because the CycleGAN network does not rely on paired real images, this color adjustment loss function can force the generator to generate an image with the same color distribution as the fog, and it can also avoid color distortion and artifacts in the generated image. The loss function of color adjustment is as follows:

(5) $$L_{Color}=\sum_{p}ANGLE(G_{A}{\left(G_{B}(y)\right)}_{p},y_{p})$$

In Formula (8), p represents one pixel; ANGLE is an angle calculation function, which calculates the angle difference between two colors. RGB can be regarded as a three-dimensional vector of colors. y means a clear image. $G_{B}(G_{A}(x))$ and $G_{A}(G_{B}(y))$ are reconstructed images. Calculate the included angle between the color vector of each pixel in $G_{A}(G_{B}(y))$ and the color vector of each pixel in image y and sum them up to obtain the color loss function.

## 4. Results

### 4.1. Experimental Data Set and Parameter Environment

The training image library used in this paper mainly trains 1500 pairs of foggy images and fogless images filtered on the D-Hazy [33] data set. The D-Hazy data set is based on the Middlebury and NYU-Depth data sets, which provide images of various scenes and their corresponding depth maps. A data set contains more than 1400 pairs of images, including the ground real reference image and the blurred image of the same scene. Moreover, NYU-Depth data set consists of video sequences of various indoor scenes recorded by Microsoft Kinect’s RGB and Depth cameras; the Middlebury data set includes indoor images of 11 scenes under many different lighting conditions and exposures (including flash and “flashlight” lighting of mobile devices). The weight factor in the loss function is 0.05. The experimental platform operating system is Windows10, the CPU is 12th Gen Intel (R) Core (TM) i7-12700, the GPU is NVIDIA RTX A5000 and the experimental development environment is Python 3.7, Pytorch 1.11.0, CUDA 11.3. The adaptive moment estimation (Adam) optimizer is used. The initial learning rate of the network is set to 0.05, the size of the trained image batch is set to 20 and the size of the trained images in the network is 256 × 256. The convolution layer inside the network uses ReLU as the activation function, and the activation function of the output layer is Sigmaid. The network is trained to 400 epochs.

In this paper, PSNR (Peak Signal to Noise Ratio) and SSIM (Structural Similarity) objective evaluation indicators are used to evaluate the image quality after defogging. PSNR can evaluate the image quality and defogging effectiveness at the pixel level. The higher the value, the better the image quality; SSIM measures image similarity from brightness, contrast and structure, respectively. The larger the value, the better the structure information that will be saved.

### 4.2. Ablation Experiment

In order to verify the effectiveness of the Dep module and the multi-head attention mechanism proposed by ADE-CycleGAN in image dehazing tasks, this paper has carried out a series of ablation experiments on the D-Hazy data set, with CycleGAN as the baseline model. The experimental results are shown in Table 1. As shown in the table, the average PSNR of ADE-CycleGAN proposed in this paper is 21.38, which is 8.1% higher than that of the baseline, indicating that the network proposed in this paper can effectively improve the defogging effect of image defogging and reduce fog. Each module discussed in this paper has improved the defogging performance of the network to a certain extent. The Dep residual block module obtains the local and global information of the image by fusing the idea of U-Net network and enhances the details of the image. The PSNR is 5.1% higher than the baseline. The introduction of the multiple attention mechanism improved the expression ability of receptive field, enhanced the features of the network and balanced the bias generated by the same attention mechanism, which made SSIM increase by 5.0% compared with the baseline. The proposed two modules increase the parameters and computation of the model to a certain extent, but they are improved in PSNR and SSIM compared with CycleGAN.

### 4.3. Performance Comparison of Each Network on Test Set

**Figure 6:**
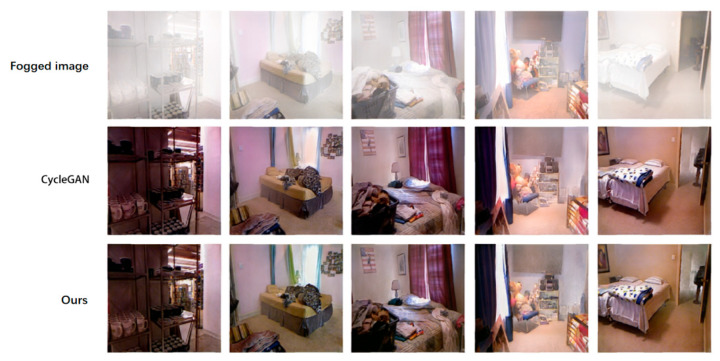
Comparison of the demisting results of CycleGAN and this method on the D-Hazy (NYU-Depth) test set.

**Figure 7:**
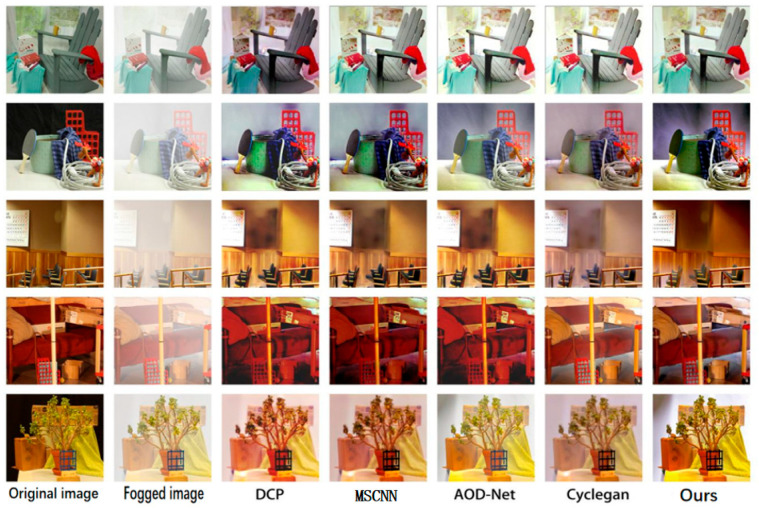
Comparison of demisting results of various methods on D-Hazy (Middlebury) test set.

**Figure 8:**
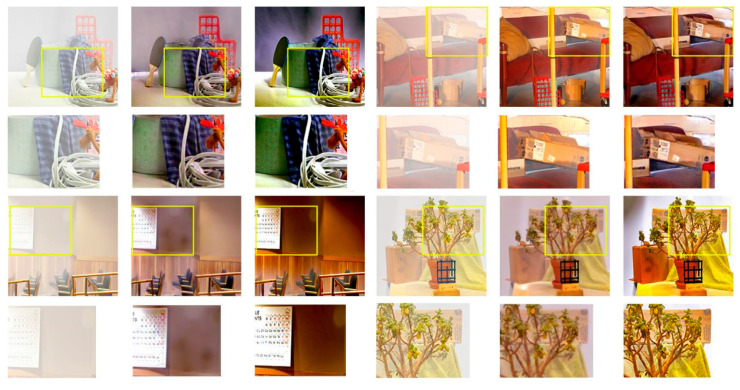
Comparison of the defogging results of this method and CycleGAN in detail.

In this paper, four mainstream defogging algorithms, DCP, MSCNN, AOD-Net and CycleGAN, are reproduced for their defogging effects on the D-Hazy data set and compared with ADE-CycleGAN in Figure 6 and Figure 7. The objective indicators for comparison are PSNR and SSIM. The comparison results are shown in Table 2. It can be seen from Table 2 that the objective indicators PSNR and SSIM of ADE-CycleGAN in the D-Hazy data set proposed in this paper are the highest. Compared with the DCP method, PSNR and SSIM are increased by 17.9% and 13.5%, respectively. Compared with the CycleGAN network, PSNR and SSIM are increased by 8.1% and 12.2%, respectively. Moreover, at the same time, in terms of subjective evaluation, the ADE-CycleGAN network can better retain the original details and color information in most images. The Figure 8 shows that the method in this paper captures more image details by introducing a feature fusion residual block and multi-head attention mechanism, and it avoids the loss of image details after defogging by the network algorithm. Secondly, the color adjustment algorithm can avoid the color difference between the defogging image and the original image, which helps to preserve the color consistency between the reconstructed image and the original image. It can moreover effectively improve the brightness of the image without affecting the defogging quality. Due to the increased depth of the network and the increased amount of calculation of network parameters, the image defogging speed is not as fast as mainstream defogging algorithms such as DCP and MSCNN. However, the objective evaluation index of this paper is the highest in terms of overall defogging effect and detail feature retention. Therefore, compared with the other methods, the ADE-CycleGAN network can have a better defogging effect in terms of the detail feature and color retention.

## 5. Discussion

We added the Dep residual connection block to the generator of the CycleGAN network and introduced a new multi-head attention mechanism. Finally, in order to avoid the change in background color, we introduced the concept of color consistency loss, improved the basic network, made the network more capable of feature extraction, preserved the overall defogging information of the image and better preserved the color information of the image after defogging. The ablation experiment shows that the improved network model has better defogging performance, but increasing the network depth also leads to longer calculation time of the model.

The improved network model and image defogging method in this paper can be used in many computer vision systems such as monitoring, intelligent transportation, civil aviation assistance, disaster relief, remote sensing observation, automatic driving, etc. For example, for community monitoring, the improved generation of the confrontation network model and the image defogging method described in this paper can effectively process the images of people entering and leaving the community in fog and haze in autumn and winter. The problem of personnel omission caused by bad weather is avoided, thus effectively improving the ability of the monitoring system to cope with bad weather.

Although the PSNR and SSIM of the ADE-CycleGAN network are both optimal after image defogging, the introduction of the multi-head attention mechanism will also increase the complexity of the network, resulting in an increase in the network’s defogging operation time. In the future, we will continue to study and improve the network to reduce its complexity.

## 6. Conclusions

To solve the problems of image detail loss and image color difference after image defogging, this paper proposes an image defogging generation network based on CycleGAN to improve the defogging quality after image reconstruction. This network combines the feature fusion idea of U-Net with the deep Dep residual block, so that the generator network can better capture the details in the image background, which is conducive to improving the defogging effect of the generation network. Moreover, the introduction of the multi-head attention mechanism can also improve the receptive field of the network, enhance the expression ability of features and balance the deviation caused by the same attention mechanism. At the same time, channel-by-channel convolution and point-by-point convolution are introduced into the Dep depth residual block to extract the deep details of the image while reducing the amount of network computing parameters. In addition, in order to solve the problem of color change after image defogging, this paper adds color adjustment loss to the loss function and gives the corresponding weight factor. By calculating the difference between the pixel points in the generated image and the original image, the generator can generate a defogging image closer to the original image. On the D-Hazy data set, ADE-CycleGAN achieves 21.38 PSNR, an 8.1% improvement over CycleGAN, while achieving 0.92 SSIM, a 12% improvement over CycleGAN, and compared with the defogging network, it has a better detail retention ability and defogging effect subjectively, and can adapt to defogging tasks under different environments. However, compared with the other network, the network structure of this paper is more complex and has a large amount of parameter calculation. In the future, more lightweight networks can be explored to achieve the effect of defogging.

## Figures and Tables

**Table 1:** Ablation experimental results.

Baseline	DEP	Attention Mechanism	PSNR	SSIM
√			19.78	0.82
√	√		20.78	0.88
√		√	20.31	0.86
√	√	√	21.38	0.92

**Table 2:** Objective parameter evaluation indicators of each network.

Method	PSNR	SSIM	Time Measure (10 Epochs/h)
DCP	18.12	0.81	3.1
MSCNN	17.87	0.73	3.1
AOD-Net	17.51	0.78	3.4
CycleGAN	19.78	0.82	3.8
Ours	21.38	0.92	4.9

## Data Availability

The data presented in this study are available on request from the corresponding author.
